# Impact of inappropriate antifungal therapy according to current susceptibility breakpoints on Candida bloodstream infection mortality, a retrospective analysis

**DOI:** 10.1186/s12879-017-2846-2

**Published:** 2017-12-06

**Authors:** María Fernanda González-Lara, Pedro Torres-González, Patricia Cornejo-Juárez, Consuelo Velázquez-Acosta, Areli Martinez-Gamboa, Andrea Rangel-Cordero, Miriam Bobadilla-del-Valle, Luis Ostrosky-Zeichner, Alfredo Ponce-de-León, José Sifuentes-Osornio

**Affiliations:** 1 Department of Infectious Diseases, Instituto Nacional de Ciencias Médicas y Nutrición Salvador Zubirán, Vasco de Quiroga 15, Belisario Domínguez Sección XVI, Tlalpan, Zip Code 14080 Mexico City, Mexico; 2 Department of Infectious Diseases, Instituto Nacional de Cancerología, Ave. San Fernando 22, Belisario Dominguez Sección XVI, Zip code 14080 Mexico City, Mexico; 3 University of Texas Health Science Center at Houston, 6431 Fannin. MSB 2.112, Houston, TX 77030 USA; 4 Department of Medicine, Instituto Nacional de Ciencias Médicas y Nutrición Salvador Zubirán, Vasco de Quiroga 15, Belisario Domínguez Sección XVI, Tlalpan, Zip Code 14080 Mexico City, Mexico

**Keywords:** Antifungal susceptibility testing, Candida bloodstream infections, Fluconazole resistance, Candidemia treatment

## Abstract

**Background:**

The mortality of Candida Bloodstream Infection (CBSI) remains high. Antifungal susceptibility breakpoints were recently updated for *Candida* species, the impact remains unknown. In this study we evaluated the impact of inappropriate antifungal treatment according to recent breakpoints on 30-day mortality of CBSI.

**Methods:**

From June 2008 to July 2014, data on CBSI episodes from two tertiary-care centers, treated > 72 h were analyzed. Antifungal therapy and 30-day mortality were registered. Inappropriate antifungal treatment according to current Clinical & Laboratory Standards Institute (CLSI) breakpoints was adjusted with 30-day mortality-related co-variates.

**Results:**

One hundred forty-nine episodes of CBSI were analyzed. The most frequent species were: *C. albicans* (40%), *C. tropicalis* (23%) and *C. glabrata* complex (20%). According to the 2012 CLSI, 10.7% received inappropriate treatment. The 30-day mortality was 38%; severe sepsis [Odds ratio (OR) 3.4; 95% CI 1.3–8.4], cirrhosis (OR 36; 95% CI 12.2–605), early central venous catheter removal (OR 0.23; 95% CI 0.08–0.66) and previous antifungal therapy (OR 0.15; 95%CI 0.03–0.62), were associated with 30-day mortality by multivariate analysis. Inappropriate antifungal treatment was not (OR 0.19; 95% CI 0.03–1.2).

**Conclusions:**

Appropriate antifungal therapy according to CLSI 2012 did not have an impact on mortality. Mortality of CBSI remains high due to disease severity and comorbidities; early antifungal therapy and catheter removal may reduce it.

## Background

*Candida* bloodstream infections (CBSI) represent 10% of all bloodstream infections. The CBSI incidence in North America reaches 0.3–0.9 cases per 1000 admissions, whereas in Latin America it may reach 5.3 cases per 1000 admissions [1–4]. The mortality rate of CBSI remains high (46%–75%) despite the availability of effective antifungal therapy [1]. Inappropriate antifungal therapy has been reported as a risk factor for increased mortality [5]. However, routine clinical care in many sites is not guided by antifungal susceptibility testing (AST) but selected according to the species-specific known susceptibility and the patient’s clinical condition.

The Clinical and Laboratory Standards Institute (CLSI) recently defined new clinical breakpoints (CBP) for the most common *Candida* species to categorize them into resistant, intermediate and susceptible and guide antifungal therapy, these breakpoints are now drug and species specific [6]. The rationale for these modifications was the relationship between a higher minimal inhibitory concentration (MIC) and worse outcome showed in some studies, epidemiologic cutoffs values, and pharmacokinetic/pharmacodynamic studies [7]. However, the impact of these modifications on mortality remains to be defined. Broth microdilution (BMD) is the reference method for *Candida* species AST; unfortunately, it is labor intensive, expensive, requires trained personnel, and is subjected to reading bias. Nowadays, antifungal susceptibility testing by automatized systems is considered reliable and may be more easily incorporated into routine clinical care [8–10].

The objective of this study was to assess the impact that the modified antifungal susceptibility breakpoints might have had on 30-day mortality of CBSI.

## Methods

Study design. A retrospective laboratory-based survey was performed; patients admitted to two referral tertiary centers in Mexico City from June 2008 to July 2014 with a positive blood culture for *Candida* spp., 30-day survival was retrieved. Socio-demographic and clinical variables were recorded in a specially designed case report form. One hundred and forty-nine samples from 149 patients who received antifungal treatment ≥ 72 h were considered for this analysis. During the study period, AST was not routinely available in the study’s centers, the selection of antifungal treatment was decided by the attending physician according to the patient’s clinical condition and the *Candida* species isolated. For this analysis, AST was performed post hoc from the available stored isolates. Our main objective was to identify whether inappropriate antifungal therapy, considering the updated susceptibility breakpoints, was associated with an increased 30-day mortality. A secondary objective was to describe the prevalence of antifungal resistance during the study period.

### Definitions

Empirical therapy was defined as the initiation of an antifungal agent at the first clinical suspicion of fungal infection [11]. Initial antifungal therapy was defined as modification of the empirical antifungal therapy or initiation of any antifungal drug after a positive blood culture with yeasts on a Gram stain, before species identification; whereas definitive treatment was the antifungal administered after species identification. Severe sepsis was defined as the presence of sepsis with any of the following: sepsis-induced hypotension, hyperlactatemia, urine output < 5 ml/Kg/h for more than 2 h following fluid resuscitation, acute lung injury with PaO_2_/FIO_2_ < 250 or < 200 in the absence and presence of pneumonia, creatinine > 2 mg/dL, bilirubin > 2 mg/dL, platelet count < 100,000 cell/mm^3^ or coagulopathy [12].

Antifungal therapy was considered appropriate if the patient received an antifungal agent for which, the *Candida* isolate was susceptible according to CLSI 2012 breakpoints and inappropriate if the MIC of the isolate was in non-susceptible range [6, 13].

### Laboratory procedures

All the *Candida* spp*.* isolates recovered from blood cultures during the study period were sent to a central laboratory for identification and AST. The isolates were cultured on Sabouraud media, underwent germ tube testing and were identified using the Vitek 2, (BioMeriéux, Lyon, France). A sample from each culture was stored as water suspension at −80 °C, until retrieved for susceptibility testing. A small aliquote was unfrozen at room temperature for 24 h, and cultured on Sabouraud agar at 30 °C for 24 h to ensure viability. AST was performed on 178 available isolates of which 149 received antifungal treatment and were included for analysis. AST was performed with Vitek 2 using the AST-YS07 card (Biomérieux, Lyon, France) and interpreted according to CLSI guidelines in document M27A3 and its updated version in M27-S4. For those isolates without a species specific clinical breakpoint, epidemiologic cutoff values were used (*C. guilliermondii, C. pelliculosa and Clavispora lusitaniae*). For species without a reported epidemiological cut off value, AST was classified according to the Vitek-2 software (Global CLSI-based + Natural Resistance) (V. 07.01) [14–16].

The reference strains *C. parapsilosis* ATCC 22019 and *C. krusei* ATCC 6258 were used as controls. Species identified as *C. parapsilosis* complex and *C. glabrata* complex refer to *C. parapsilosis* complex and *C. glabrata* complex, since phenotypic testing cannot differentiate the cryptic species within the complexes. Isolates were considered susceptible to echinocandins if the micafungin MIC was within the susceptible ranges, since anidulafungin is not available for YS07 card, and the known variability in the caspofungin MIC with Vitek2 [17, 18]. Assays to identify antifungal resistance mechanisms were not done.

### Statistical analysis

Categorical data was summarized using frequency tables, and the χ^2^ test was used for comparison between groups. Characteristics of patients were compared using Mann-Whitney U test for continuous variables. To evaluate 30-day mortality in multivariate analysis, we built a multiple logistic regression model including inappropriate antifungal treatment as well as variables with a *p*-value < 0.2 in univariate analysis or biological plausibility. Stratification for severe sepsis is provided in a separate analysis. Odds ratio (OR) with 95% confidence interval (CI) was reported. A *p*-value < 0.05 was considered significant for all the analysis. Stata 11.0 (Stata Corp College Station, TX) software was used for analysis.

### Ethics statement

The study was reviewed and approved by the Scientific and Bioethics Committee at the Instituto Nacional de Cancerología (reference number INCAN/005/10) and the Research and Bioethics Committee at the Instituto Nacional de Ciencias Médicas y Nutrición “Salvador Zubirán” (reference number 1912). Because of the observational nature of the study, a waiver of informed consent was granted. Authors involved in data analysis could not identify individual patients since the database used numbers specific to the study.

- Availability of data and materials. The datasets used and/or analyzed during the current study are available from the corresponding author on reasonable request.

## Results

**Fig. 1:**
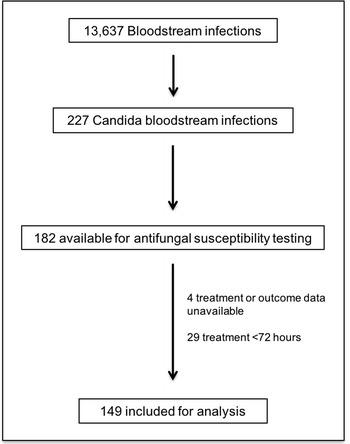
Flowchart of patients during the study period

During the 72 months of the study period, 83,942 blood cultures were collected in both institutions; 16.2% (13,637) yielded a positive result, and 1.7% (227) corresponded to episodes of CBSI. One hundred and seventy-eight isolates (78.4%) were available for AST at the end of the study period. Of these, 84% (149/178) were episodes treated for ≥ 72 h and were included for analysis (Fig. 1).

At the time of CBSI diagnosis, 25% (37/149) of the patients were admitted to intensive care unit (ICU), and 75% (112/149) were admitted to medical or surgical wards. Thirty percent (45/149) had diagnosis of hematologic malignancy and 17.4% (26/149) other forms of cancer. APACHE II score, to assess mortality and disease severity was available for 122 episodes with a median score of 18.9 (IQR 14–24).

The possible source of the CBSI was identified in 49% (73/149) of the episodes, 30.8% (46/149) had an intra-abdominal infection, 7.3% (11/149) catheter-relatedinfection, and 10.7% (16/149) had both*.* Ten percent (16/149) were diagnosed with additional forms of candidiasis (n: 5 hepatosplenic candidiasis, n: 4 skin involvement, n: 3 endocarditis, n: 2 ophthalmic candidiasis, n: 2 osteomyelitis). Forty-four percent developed severe sepsis, and 22% (34/149) had neutropenia at CBSI diagnosis of which, 17.6%% (6/34) received primary antifungal prophylaxis. Eighty-two percent (123/149) had a central venous catheter in place at the time of CBSI diagnosis. Early central venous catheter (CVC) removal (< 72 h) was achieved in 59% (73/123) of the episodes (Table 1).

**Table 1:** Thirty-day mortality in Candida bloodstream infection. Univariate analysis

	Total, *n* = 149	30-d Survivors, *n* = 93	30-d Non-survivors *n* = 56	*p*-value
Demographics				
Female	78 (52.3)	50 (53.7)	28 (50)	0.65
Age, median (IQR)	47 (32–58)	44 (30–55)	48 (35.5–61.5)	0.23
Intensive care unit admission	37 (24.8)	15 (16.1)	22 (39.2)	< 0.001
APACHE II *n* = 122, median (IQR)	18 (14–24)	16 (12–20)	23 (18.5–28.5)	< 0.001
Severe sepsis	66 (44.3)	29 (31.1)	37 (66)	< 0.001
Comorbidities				
Hematologic neoplasia	45 (30.2)	22 (23.6)	23 (41)	0.025
Solid neoplasia	26 (17.4)	17 (18.2)	9 (16)	0.73
Cardiovascular	37 (24.8)	22 (23.6)	15 (26.7)	0.7
Chronic kidney disease	18 (12)	10 (10.7)	8 (14.2)	0.52
Cirrhosis	11 (7.3)	1 (1)	10 (17.8)	< 0.001
Diabetes mellitus	19 (12.7)	12 (12.9)	7 (12.5)	0.94
Intra-abdominal infection	62 (41.6)	45 (48.3)	17 (30.3)	0.031
Neutropenia	34 (22.8)	16 (17.2)	18 (32.1)	0.035
Cancer CT	38 (25.5)	18 (19.3)	20 (35.7)	0.026
CA-BSI	27 (18.1)	22 (23.6)	5 (8.9)	0.024
CVC	123 (82.5)	80 (86)	43 (76.7)	0.15
Early CVC removal	73 /123 (59.3)	55/80 (68.7)	18 /43 (41.8)	0.004
Total parenteral nutrition	49 (32.8)	36 (38.7)	13 (23.2)	0.034
Empiric antifungal therapy	35 (23.8)	27 (29.6)	8 (14.3)	0.033
Steroid use	40 (26.8)	20 (21.5)	20 (35.7)	0.08
Time to antifungal therapy median (IQR)	2 (1–3)	2 (1–2)	2 (1–3)	0.165
Initial treatment				
Echinocandin	64 (42.9)	42 (45.1)	22 (39.1)	0.48
Caspofungin	43 (28.8)	27 (29)	16 (28.5)	0.89
Anidulafungin	21 (14.1)	15 (16.1)	6 (11.3)	0.4
AmB-d	61 (40.9)	35 (37.6)	26 (46.4)	0.29
Azoles	25 (16.7)	16 (17.2)	9 (16)	0.85
Fluconazole	24 (16.1)	15 (16.1)	9 (16)	0.95
Voriconazole	1 (0.6)	1 (1)	–	–
Definitive treatment				
Echinocandin	56 (37.5)	36 (38.7)	20 (35.7)	0.71
Caspofungin	31 (20.8)	17 (18.2)	14 (25)	0.34
Anidulafungin	25 (16.7)	19 (20.4)	6 (10.7)	0.23
AmB-d	46 (30.8)	21 (22.5)	25 (44.6)	0.005
Azoles	47 (31.5)	36 (38.7)	11 (19.6)	0.015
Fluconazole	41 (27.5)	31 (33.3)	10 (17.8)	0.4
Voriconazole	6 (4)	5 (5.3)	1 (1.8)	0.27
Inappropriate initial antifungal treatment	13 (8.7)	9 (9.6)	4 (7.1)	0.59
Inappropriate definitive antifungal treatment	8 (5.3)	6 (6.4)	2 (3.5)	0.36
Inappropriate initial and/or definitive treatment	16 (10.7)	11 (11.8)	5 (8.9)	0.58
Non-albicans CBSI	89 (59.7)	62 (66.6)	27 (48.2)	0.026
*C. tropicalis*	35 (24)	19 (20)	16 (29)	0.25
*C. glabrata*	28 (19)	19 (20.4)	9 (16)	0.5
*C. parapsilosis*	16 (10.7)	15 (16)	1 (1.8)	0.006

*IQR* interquartile range, *APACHE* acute physiology and chronic health evaluation, *CT* chemotherapy, *CA-BSI* catheter associated blood stream infection, *CVC* central venous catheter, *AmB-d* amphotericin B-deoxycholate, *CBSI* candida blood stream infection

### *Candida* species distribution and antifungal susceptibility

Blood cultures yielded a positive result for *Candida* spp. in a median of 2 days (IQR 1–3) after drawn. The most frequent isolated species were: *C. albicans* (n: 60, 40%), *C. tropicalis* (n: 34, 23%), *C. glabrata* complex (n: 30, 20%), *C. parapsilosis* complex (n: 15, 10%) and *C. guilliermondi* (n: 3, 2%). Other less frequent were: *C. krusei* (n: 2, 1.3%), *C. norvegensis* (n: 1, 0.6%), *C. inconspicua* (n: 1, 0.6%), *C. lusitaniae* (n:1, 0.6%) *C. lipolytica* (n: 1, 0.6%) and *C. pelliculosa* (n: 1, 0.6%).

According to the 2012 CLSI susceptibility breakpoints, 70% (104/149) of the isolates were susceptible to fluconazole, 95.3% (142/149) to caspofungin and, 97.3% (145/149) isolates to micafungin; and 98.6% (147/149) were susceptible to AmB (Table 2).

**Table 2:** Antifungal susceptibility of 149 isolates and categorical agreement between CLSI 2008 and 2012

Species/ antifungal (n)	MIC range (mg/L)	MIC _50_ (mg/L)	MIC _90_ (mg/L)	CLSI 2008 n (%)			CLSI 2012 n (%)		
				S	I	R	S	SDD^a^/I	R
*C. albicans* (60)									
Fluconazole	≤ 1–8	1	1	60 (100)	–	–	56 (93.3)	2 (3.3)	2 (3.3)
Voriconazole	≤ 0.12	0.12	0.12	60 (100)	–	–	60 (100)	–	–
Caspofungin	≤ 0.25–1	0.25	0.25	60 (100)	–	–	56 (93.3)	1 (1.6)	3 (5)
Micafungin	≤ 0.06–1	0.06	0.06	60 (100)	–	–	58 (96.6)	1 (1.6)	1 (1.6)
*C. tropicalis* (34)									
Fluconazole	≤ 1–8	1	1	34 (100)	–	–	32 (94.1)	1 (2.9)	1 (2.9)
Voriconazole	≤ 0.12	0.12	0.12	34 (100)	–	–	34 (100)	–	–
Caspofungin	≤ 0.25–1	0.25	0.25	34 (100)	–	–	34 (100)	–	–
Micafungin	≤ 0.06–0.5	0.06	0.06	34 (100)	–	–	34 (100)	–	–
*C. glabrata* (30)									
Fluconazole	≤ 1–64	4	8	28 (93.3)	–	2 (6.6)	0	28 (93.3)	2 (6.6)
Caspofungin	< 0.25–1	0.25	0.25	30 (100)	–	–	29 (96.6)	–	1 (3.3)
Micafungin	< 0.06–0.5	0.06	0.06	30 (100)	–	–	29 (96.6)	–	1 (3.3)
*C. parapsilosis* (15)									
Fluconazole	≤ 1–8	1	8	15 (100)	–	–	13 (86.6)	1 (6.6)	1 (6.6)
Voriconazole	≤ 0.12–0.25	0.12	0.12	15 (100)	–	–	15 (100)	–	–
Caspofungin	< 0.25–1	1	1	15 (100)			15 (100)	–	–
Micafungin	< 0.06–1	0.5	1	15 (100)			15 (100)	–	–
*C. guilliermondi* (3)									
Fluconazole	2–8	2	8	3 (100)			–	–	–
Voriconazole	< 0.12	< 0.12	< 0.12	3 (100)			–	–	–
Caspofungin	0.25–1	0.25	1	3 (100)			–	–	–
Micafungin	0.5	0.5	0.5	3 (100)			–	–	–
*C. kruzei* (2)									
Voriconazole	≤ 0.12	0.12	0.12	2 (100)			2 (100)	–	–
Caspofungin	≤ 0.25–0.5	0.25	0.5	2 (100)			1	1	–
Micafungin	0.12	0.12	0.12	2 (100)			2	–	–
Other^b^ (5)									
Fluconazole	≤ 1–16	2	16	3 (60)	2 (40)		–	–	–
Voriconazole	≤ 0.12	0.12	0.12	5 (100)	–	–	–	–	–
Caspofungin	≤ 0.25- > 4	0.25	4	4 (80)	–	–	–	–	–
Micafungin	< 0.06- > 4	0.12	4	4 (80)	–	–	–	–	–

*MIC* minimal inhibitory concentration, *S* susceptible, *I* intermediate, *R* resistant, *SDD* susceptible dose dependent, *CLSI* Clinical Laboratory Standards Institute

^a^SDD applies for fluconazole and voriconazole instead of intermediate

^b^Other species: *C. lusitaniae, C. pelliculosa, C. norvegensis, C. lypolitica, C. inconspicua*

Overall fluconazole resistance was 6% (2 *C. albicans*, 2 *C. glabrata* complex, 2 *C. kruzei*, 1 *C. parapsilosis* complex, 1 *C. tropicalis* and 1 *C. pelliculosa*). Overall echinocandin resistance was 1.3% (1 *C. albicans* and 1 *C. glabrata*).

A single echinocandin-resistant *C. glabrata* was identified, which also was intermediate to fluconazole. One isolate of *C. albicans* showed resistance to every antifungal class (azoles, echinocandins and amphotericin B), from a patient who subsequently developed *C. glabrata* BSI secondary to a diabetic foot osteomyelitis in a 42-year-old male patient who died after 22 days of treatment with caspofungin.

### Antifungal treatment

Thirty-five of 149 patients (23%) were receiving antifungal therapy at the time the CBSI was identified, in 23/35 (66%) cases the indication was empiric therapy for suspected invasive fungal infection, 15/35 (42.8%) cases were receiving antifungal therapy for another demonstrated fungal infection; 18/35 (51.4%) patients received azoles (2 voriconazole and 16 fluconazole), 9/35 (25.7%) received AmB-d and 9/35 (25.7%) received echinocandins (3 caspofungin and 6 anidulafungin). In 7/35 (20%) cases, the CBSI agent was non-susceptible to empiric therapy.

Antifungal was initiated or modified within a median of two days (IQR 1–3) after the blood culture was drawn. Echinocandins were the most frequent initial treatment (42.9%), followed by amphotericin B-deoxycholate (AmB-d) (40.2%), and fluconazole (16.1%). One patient received combination therapy with AmB-d and fluconazole, and one received voriconazole. The initial antifungal agent was modified in 37% (55/149) of the episodes. Among these 47.3% (26/55) were switched to fluconazole after species identification. Twenty-three percent (13/55) were switched to other antifungal due lack of a clinical response; 12.7% (7/55) due to a nonsusceptible isolate (according to the known species-specific susceptibility); 5.4% (3/55) due to toxicity and 5.4% (3/55) due to proven or probable invasive aspergillosis; 3.6% (2/55) to treat other molds and 3.6% (2/55) due to unspecified reasons. Definitive antifungal treatment of 149 episodes was administered with echinocandins (37.5%), AmB-d (30.2%), and fluconazole (31.5%). One patient received a combination of AmB-d and fluconazole. Among the 89 non-albicans CBSI episodes, 41 (46%) received echinocandins as definitive treatment, 29 (32%) AmB-d, and 19 (21.3%) azoles. The median time of antifungal therapy was 14 days (IQR 5–20).

Regarding the CLSI 2012 breakpoints, 8.7% (13/149) patients received inappropriate initial antifungal treatment, four of which died: one patient received 12 days of fluconazole (6 mg/Kg) for a non-susceptible fluconazole *C. albicans* isolate, was switched later to caspofungin due lack of clinical response and died a day after. Two patients received fluconazole for susceptible dose-dependent (SDD) *C. glabrata;* one died five days after CBSI diagnosis, the other was switched to caspofungin on the sixth day and died. The remainder patient had a CBSI due to a fluconazole-resistant *C. albicans* isolate, the patient was started on fluconazole, was switched to caspofungin twelve days later and died 26 days after the CBSI diagnosis. Inappropriate definitive antifungal treatment was administered in 5.3% (8/149) of the episodes (5 *C. glabrata,* 2 *C. albicans,* and 1 *C. tropicalis*), two of which died: one patient received eight days of AmB-d, was switched to fluconazole for a fluconazole –SDD *C. glabrata* and died 22 days after diagnosis.

No patient received inappropriate initial or definitive treatment according to 2008 guidelines (Table 1).

### Outcome

Thirty-day mortality was 38% (56/149); non-survivors more often presented with severe sepsis and required mechanical ventilation. Non-survivors were also more likely to have hematologic neoplasia, cirrhosis, severe neutropenia and to receive definitive treatment with AmB-d. Survivors were more likely to have an intraabdominal infection, an early central-venous catheter removal, have received empirical antifungal therapy, to receive definitive treatment with azoles and to have a Non-albicans CBSI (Table 1). In multivariate analysis, severe sepsis (OR 3.4; 95% CI 1.3–8.4) and previous diagnosis of cirrhosis (OR 36 95% CI 12.2–605) were independently associated with increased 30-day mortality. Early CVC withdrawal (OR 0.23; 95% CI 0.08–0.66) and empirical antifungal therapy (OR 0.15; 95% CI 0.03–0.62) were independently associated with reduced 30-day mortality. When stratified by severe sepsis, survivors were more likely to receive empirical antifungal therapy (OR 0.05; 95% CI 0.005–0.55) and early CVC removal (OR 0.07; 95% CI 0.017–0.28) among patients without severe sepsis. Of note neither empirical antifungal therapy nor early CVC removal were associated with reduced mortality in patients with severe sepsis. Inappropriate initial or/and definitive antifungal treatment was not associated with 30-day mortality (OR 0.19; 95% CI 0.03–1.2), not even after stratification for severe sepsis (Table 3).

**Table 3:** Thirty-day mortality in Candida bloodstream infection. Multivariate analysis

Characteristic	OR (95%CI)	OR (95%CI) without severe sepsis
Age	1.01 (0.98–1.03)	1.02 (0.99–1.05)
Inappropriate antifungal treatment ^a^	0.16 (0.02–1.3)	0.1 (0.009–1.05)
Severe sepsis	3.5 (1.4–8.9)	–
Cirrhosis	42.1 (2.3–744)	56 (0.06–47,908)
Early CVC withdrawal	0.22 (0.07–0.63)	0.07 (0.017–0.28)
Empirical antifungal therapy	0.15 (0.03–0.62)	0.05 (0.005–0.55)

*OR* Odds ratio; 95% CI: 95% confidence interval

^a^According to CLSI 2012, *CT* Chemotherapy, *CVC* central venous catheter

Persistent candidemia occurred in 21% (34/149). Inappropriate treatment was not associated with increased frequency of persistent candidemia (*p* = 0.7).

## Discussion

In this observational study, we did not find an association between inappropriate antifungal therapy defined by the updated CLSI clinical breakpoints and increased mortality. Of note, severe sepsis, and cirrhosis were independently associated with increased mortality. On the contrary, modifiable factors such as empirical antifungal therapy and early CVC removal, were independently associated with decreased 30-day mortality.

The rationale of the CLSI 2012 updates was to identify the emergence of acquired mechanisms of resistance promptly. Recently IDSA treatment guidelines have advocated the testing for azoles to all bloodstream and other relevant isolates for testing, as well as echinocandin testing for those cases with previous exposure and species with known emerging resistance to these drugs, when previous expert recommendations suggested AST only when a patient was not improving [15, 19–21]. However, it is not clear if AST for CBSI should be routinely performed and the impact of routine AST for CBSI on mortality is not clear [22, 23].

In a similar study, in which AST by automatized systems (Sensititre YeastOne) was interpreted according to previous CBP, appropriate early treatment was not associated with survival in multivariate analysis. However, in a subgroup analysis excluding patients who received < 24 h of appropriate antifungal therapy, the authors found a statistically significant association between survival and appropriate-early treatment [24]. No other study has reported the impact of updated breakpoints on clinical outcome. In this study, we excluded patients with less than 72 h of antifungal treatment and did not find improved survival among those patients who received appropriate antifungal therapy. However, our definition of appropriateness did not include the dosage of antifungals; this issue becomes more critical while evaluating azoles, which require weight-adjusted loading dose. Contrary to what was expected, the azole therapy showed a tendency to increased survival by univariate analysis, whereas the opposite was observed for AmB-d. This may be explained by the fact that a larger proportion of critical patients received fungicidal treatment rather than fluconazole.

After stratifying for severe sepsis, we identified variables associated with 30-day mortality for CBSI: Cirrhosis, a risk factor for infection and increased mortality due to the multifactorial immune dysfunction occurring in these patients [25–28]. and the removal of a CVC. While several retrospective analysis show increased survival and reduced duration of the CBSI when removing the CVC [29–31]. other studies have not supported these findings [32]. No trial has randomized catheter withdrawal, but a recent patient level quantitative review of 1915 patients from 7 randomized trials confirmed that CVC removal at any time during treatment was associated with a reduced 30-day mortality, clinical and microbiological succes. In our study, this finding remained significant in both, the *C. albicans* and the non-albicans *Candida* subgroups, as well as in the lowest APACHE II score quartiles [33]. Furthermore, empirical antifungal therapy was independently associated with reduced 30-day mortality. It has been previously demonstrated that mortality increases wih each subsequent day that empirical therapy is delayed [34, 35].

We found an important proportion of CBSI caused by non-albicans species, the increasing prevalence of non-albicans CBSI has been previously reported worldwide. [3, 36] The increasing rate of isolation of *C. glabrata* in Brazil and Latin America may be related to older age, geographic factors, and underlying comorbidities such as cancer, particularly in cases not related to selective pressure with fluconazole or areas with low fluconazole resistance rates; *C. parapsilosis* is related to indwelling catheters and chronic conditions and *C. tropicalis* usually occurs among patients with hematological malignancies, neutropenia and mucositis; all of which are risk factors frequently found in tertiary care centers, as in this study [1].

In this study, most *C. albicans* isolates remained susceptible to fluconazole and to echinocandins when tested through the automatized system Vitek2. These results agree with a recent report from the SENTRY study using the updated CBPs by BMD [37], and within the range reported by other studies [38–40]. Resistance to fluconazole or echinocandins among *C. glabrata* was an infrequent event, as compared to others [41].

This study has several limitations: This was an observational study thus, there was probable bias in the selection of antifungals by the attending physicians according to disease severity, hindering to predict mortality; the antifungal doses were not systematically registered, which is a sensitive issue regarding azoles; we were unable to retrieve all *Candida* isolates for performing AST, which was achieved using Vitek 2, instead of the standard of reference broth microdilution (BMD). However, AST by automatized systems is FDA approved and considered to be comparable to BMD, with the benefit of being a standardized, reproducible, easier to perform and less expensive method [8–10, 42].

## Conclusion

Inappropriate antifungal treatment according with the current clinical breakpoints was not associated with mortality in this retrospective analysis. Mortality in CBSI remains high due to disease severity and comorbidities such as cirrhosis at the time of diagnosis. In addition, we identified some modifiable factors as early antifungal therapy and catheter removal which may improve the outcome of these patients. Consequently, prospective studies are needed.
